# Opening up new niche dimensions: The stoichiometry of soil microarthropods in European beech and Norway spruce forests

**DOI:** 10.1002/ece3.10122

**Published:** 2023-05-22

**Authors:** Lara Warnke, Dietrich Hertel, Stefan Scheu, Mark Maraun

**Affiliations:** ^1^ JFB Institute of Zoology and Anthropology University of Göttingen Göttingen Germany; ^2^ Albrecht von Haller Institute for Plant Sciences, Plant Ecology and Ecosystems Research University of Göttingen Göttingen Germany; ^3^ Centre of Biodiversity and Sustainable Land Use University of Göttingen Göttingen Germany

**Keywords:** diversity, elemental composition, mites, niche differentiation, soil animals, stable isotopes

## Abstract

Niche theory fundamentally contributed to the understanding of animal diversity. However, in soil, the diversity of animals seems enigmatic since the soil is a rather homogeneous habitat, and soil animals are often generalist feeders. A new approach to understand soil animal diversity is the use of ecological stoichiometry. The elemental composition of animals may explain their occurrence, distribution, and density. This approach has been used before in soil macrofauna, but this study is the first to investigate soil mesofauna. Using inductively coupled plasma optic emission spectrometry (ICP‐OES), we analyzed the concentration of a wide range of elements (Al, Ca, Cu, Fe, K, Mg, Mn, Na, P, S, Zn) in 15 soil mite taxa (Oribatida, Mesostigmata) from the litter of two different forest types (beech, spruce) in Central Europe (Germany). Additionally, the concentration of carbon and nitrogen, and their stable isotope ratios (^15^N/^14^N, ^13^C/^12^C), reflecting their trophic niche, were measured. We hypothesized that (1) stoichiometry differs between mite taxa, (2) stoichiometry of mite taxa occurring in both forest types is not different, and (3) element composition is correlated to trophic level as indicated by ^15^N/^14^N ratios. The results showed that stoichiometric niches of soil mite taxa differed considerably indicating that elemental composition is an important niche dimension of soil animal taxa. Further, stoichiometric niches of the studied taxa did not differ significantly between the two forest types. Calcium was negatively correlated with trophic level indicating that taxa incorporating calcium carbonate in their cuticle for defense occupy lower trophic positions in the food web. Furthermore, a positive correlation of phosphorus with trophic level indicated that taxa higher in the food web have higher energetic demand. Overall, the results indicate that ecological stoichiometry of soil animals is a promising tool for understanding their diversity and functioning.

## Graphical abstract

Soil mite taxa differed in their stoichiometry, indicating the existence of multidimensional stoichiometric niches in soil mites. Litter stoichiometry as well as stoichiometric niches between the two forest types did not differ pointing to limited elemental niche plasticity. Negative correlation between Ca and trophic level of soil mites indicates increased skeletal hardening as a defense in taxa low in the food web and positive correlation between phosphorus and trophic level indicates higher energetic demand of taxa high in the food web.

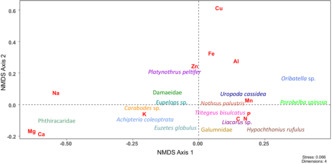

## 1 INTRODUCTION

Understanding the mechanisms that generate and maintain animal diversity is among the greatest challenges in ecology. In soil systems, animal diversity is exceptionally high, in particular at small spatial scales it may exceed that of aboveground animal communities. This is surprising considering the rather uniform basal resources, that is, dead organic matter. Explaining niche differentiation in soil systems, therefore, poses a major challenge as phrased by Anderson (1975) in his seminal paper titled “The enigma of soil animal species diversity.”

The concept of the ecological niche plays a central role in explaining species richness, distribution, and coexistence as well as the generation of species richness. Niche differentiation in soil microarthropods has been little studied. Using stable isotopes (^15^N, ^13^C) the trophic niche of soil animal species, reflecting a major component of the ecological niche of a species, has been studied (Klarner et al., 2014; Scheu & Falca, 2000; Schneider et al., 2004). In stable isotope analyses, δ^15^N values reflect the trophic position of species and δ^13^C values reflect the use of basal resources (Maraun et al., 2023; Potapov et al., 2019). Further, neutral lipid fatty acid analysis is increasingly used for investigating the trophic niche of soil animal species (Maraun et al., 2020; Ruess et al., 2007; Ruess & Chamberlain, 2010). The results suggest that trophic niches of soil arthropods are rather independent of the forest type they inhabit and also of the litter horizon they are living in (Lu et al., 2022; Scheu & Falca, 2000; Schneider et al., 2004). However, being based on two dimensions only, stable isotope analysis only displays a fraction of the niche dimensions of animal species.

Providing a wider range of dimensions, the stoichiometry of organisms is a promising tool for the characterization of the niches of species (González et al., 2017; Peñuelas et al., 2019; Reichle et al., 1969; Sardans et al., 2021; Zhang, Chen, Deng, Li, Chen, et al., 2022; Zhang, Chen, Deng, Li, González, & Wang, 2022). The method is based on the fact that organisms differ in their stoichiometry across taxonomic as well as trophic groups (Andrieux et al., 2020; González et al., 2018; Zhang, Chen, Deng, Li, Chen, et al., 2022; Zhang, Chen, Deng, Li, González, & Wang, 2022) and face stoichiometric constraints due to their individual nutritional demands (González et al., 2017; Sterner & Elser, 2002). For heterotrophic organisms, intraspecific variability is generally assumed to be small due to the necessity to maintain stoichiometric homeostasis (Andrieux et al., 2020; Persson et al., 2010). Most studies on the stoichiometry of organisms focused on the three macroelements C, N, and P, present in all organisms (Buchkowski & Lindo, 2021; González et al., 2011, 2017; Halvorson et al., 2017). However, there are about 25 relevant bioelements in animals, which comprise the biogeochemical niche of an organism (Frausto da Silva & Williams, 2001; Kaspari, 2021; Sterner & Elser, 2002). Investigating stoichiometry beyond C, N, and P, therefore, is likely to allow more detailed characterization of the stoichiometric niche of animal species (Zhang, Chen, Deng, Li, Chen, et al., 2022; Zhang, Chen, Deng, Li, González, & Wang, 2022). The stoichiometry of soil microarthropods, however, has not been investigated and therefore it remains unknown if the demand for different elements contributes to the niche differentiation of microarthropod species.

In our study, we investigated the stoichiometry of 15 soil mite taxa from European (Germany) beech and spruce forest stands regarding the following elements: aluminum (Al), calcium (Ca), carbon (C), copper (Cu), iron (Fe), magnesium (Mg), manganese (Mn), nitrogen (N), phosphorus (P), potassium (K), sulfur (S), and zinc (Zn). Additionally, we investigated δ^15^N and δ^13^C stable isotope values to evaluate trophic niches of these mite taxa in beech and spruce forest stands. We hypothesized that (1) stoichiometry differs between mite taxa due to their different nutritional requirements, (2) stoichiometry of the mite taxa occurring in both forest types does not differ as animals can extract required nutrients from different litter types, and (3) stoichiometry of mite taxa reflects their trophic position with predatory taxa containing more P, and low trophic level taxa containing more Ca in order to harden their cuticle as a defense mechanism.

## 2 MATERIALS AND METHODS

### 2.1 Study site and sample collection

Sampling took place at four beech (*Fagus sylvatica*) and four spruce (*Picea abies*) forest stands in the Hainich‐Dün forest, a large beech‐dominated mountain range in central Germany. One bag of litter from an area of ~3 m^2^ was taken at each forest stand in autumn 2021. To avoid spatial autocorrelation, the stands were between 500 m and 10 km apart from each other. Typical understory plant species at our forest sites include *Anemone nemorosa*, *Allium ursinum*, *Leucojum vernum*, and *Mercurialis perennis*, with these species being more prominent in beech than in spruce forest stands. The Hainich is based on calcareous bedrock partly covered by Pleistocene loess deposits, soils are mainly luvisols. The pH values (in H_2_O) are ranging between 5.0 and 6.0. The average annual precipitation is ~650 mm (Fischer et al., 2010; www.biodiversity‐exploratories.de).

### 2.2 Extraction and measurements of elemental content

Animals were extracted using heat (Kempson et al., 1936) and collected in tap water alive. In total, 15 mite taxa were collected (*Achipteria coleoptrata*, *Carabodes* sp., Damaeidae, *Eupelops* sp., *Euzetes globulus*, Galumnidae, *Hypochthonius rufulus*, *Liacarus* sp., *Nothrus palustris*, *Oribatella* sp. Phthiracaridae, *Platynothrus peltifer*, *Porobelba spinosa*, *Tritegeus bisulcatus*, *Uropoda cassidea*). The animals were collected and determined alive using Weigmann (2006) for oribatid mites and Karg (1989) for Uropodina and subsequently frozen at −20°C. For measuring element concentrations, the animals were dried at 70°C for 48 h and weighed into digestion vials; 20 to 60 animals were pooled to reach a minimum weight of 0.3 mg for ICP‐OES analysis. Samples from the organic litter layer were dried at 70°C for 48 h, milled, and weighed into digestion vials. For each taxon (if present) and for litter, one measurement was performed for each site. Two milliliters of nitric acid (65%) were added to the vials for liquid digestion (185°C, for 7 h). Element concentrations were analyzed using inductively coupled plasma optic emission spectrometry (ICP‐OES; Thermo Fisher Scientific iCAP 7000) and included Al, Ca, Cu, Fe, K, Mg, Mn, Na, P, and Zn. NIST‐SRM ‘1547 Peach Leaves’ (50 mg; National Institute of Standards and Technology, Standard Reference Material) was used as an internal standard (www.nist.gov).

For measuring C and N concentration as well as ^15^N/^14^N and ^13^C/^12^C ratios, samples were analyzed using an elemental analyzer (NA 1110, CE‐Instruments) coupled with an isotope ratio mass spectrometer (MAT 251, Finnigan; Reineking et al., 1993) at the Centre for analyses of stable isotopes (KOSI), University of Göttingen. Stable isotope ratios were measured using the *δ* notation as *δX* (‰) = (*R*_sample_ − *R*_standard_)/*R*_standard_ × 1000, with *R* being the ratio between the heavy and light isotopes (^13^C/^12^C or ^15^N/^14^N) in the sample and standard, respectively. Vienna PeeDee Belemnite was used as a standard for δ^13^C and nitrogen in atmospheric air for δ^15^N. The internal standard was acetanilide. We calibrated δ^15^N and δ^13^C signatures to beech and spruce litter, respectively, and denoted them as Δ^15^N and Δ^13^C values.

### 2.3 Statistical analyses

All analyses were carried out using R 4.0.3 (R Core Team, 2020). Prior to the analyses, sulfur (S) was excluded as concentrations often were below the detection limit. We performed a variance partitioning analysis to estimate the proportion of explained variance for the main factors, that is, “Taxon” and “Forest type” (beech, spruce) (varpart(), R package vegan; Oksanen et al., 2022). The concentrations of C, N, P, K, Na, Ca, Mg, Fe, Mn, Cu, Al, and Zn of spruce and beech litter were investigated by non‐metric multidimensional scaling (NMDS; metaMDS(), R package vegan) with Pillai's Trace as distance matrix. This resulted in a dataset with two dimensions (dimcheckMDS(), R package goeveg; Goral & Schellenberg, 2021; stress value = 0.082). Subsequently, the values of the two NMDS axes were used as dependant variables in a multivariate analysis of variance (MANOVA; manova() R package stats; R Core Team, 2020). “Forest type” was used as a fixed factor.

Element concentrations of the ten mite taxa which occurred in both spruce and beech forests (*Achipteria coleoptrata*, *Carabodes* sp., Damaeidae, *Eupelops* sp., Galumnidae, *Liacarus* sp., *Oribatella* sp., Phthiracaridae, *Platynothrus peltifer*, *Uropoda cassidea*) were also analyzed by NMDS (see above). This resulted in a dataset with four dimensions (stress value = 0.066), which was analyzed by MANOVA using “Forest type” as a fixed factor. Further, element concentrations of all 15 studied mite taxa (see above) were investigated by NMDS (see above). This resulted in a dataset with four dimensions (stress value = 0.066), which was analyzed by MANOVA with “Taxon” as a fixed factor. “Forest type” was excluded as a factor as it was not significant; see below.

Using the NMDS data, a post hoc multivariate pairwise comparison was carried out (Hotellings *T* ^2^ test; hotellingsT2(), R package ICSNP; Nordhausen et al., 2018) to inspect differences in element composition between each of 13 mite taxa. *E. globulus* and *P. spinosa* were excluded from this analysis since only two replicates of these species were measured.

Finally, we inspected which elements were responsible for significant MANOVA results, i.e. which elements differed significantly between the mite taxa using separate ANOVAs (anova, R package stats (R Core Team, 2020)) with “Taxon” as a fixed factor and the concentrations of each element as the dependant variable. Tukey's post hoc pairwise multiple comparison was used to identify mite taxa differing in element concentrations.

To inspect correlations between Δ^15^N values and the 12 studied elements, linear regressions were performed (lm(), R package stats; R Core Team, 2020). Before the analyses, the data of Phthiracaridae were excluded from the dataset as their low C and N concentrations dominated the analysis. Including them in a linear regression would have resulted in two clouds of datapoints precluding the use of regression analysis.

## 3 RESULTS

The stoichiometry of beech and spruce litter did not differ significantly (MANOVA; Pillai's Trace = 0.11, approx. *F*_2,5_ = 0.31, *p* = .745). Also, the stoichiometry of the 10 mite taxa which occurred in both the spruce and beech forests did not differ between the two forest types (MANOVA; Pillai's trace = 0.04, approx. *F*_4,58_ = 0.67, *p* = .61). Variance partitioning analysis revealed that 40.4% of the variation was explained by taxon, and only 2.0% by forest type (based on adj. *R* ^2^). Therefore, the factor ‘Forest type’ was excluded from further analyses allowing to compare the stoichiometry of all the 15 mite taxa.

**FIGURE 1:**
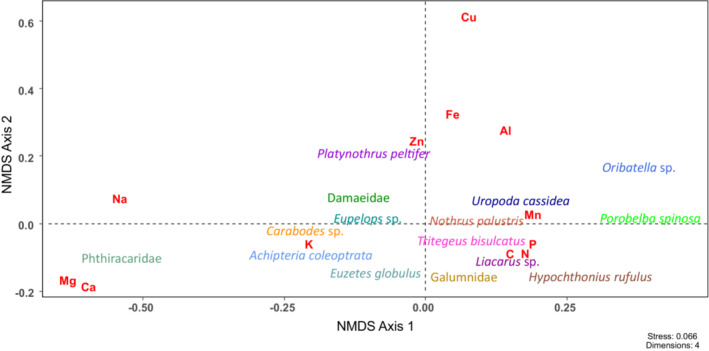
Elemental composition of the investigated mite taxa as reflected by the first two axes of NMDS of elemental concentrations. Mites of the beech and spruce forest are pooled.

**FIGURE 2:**
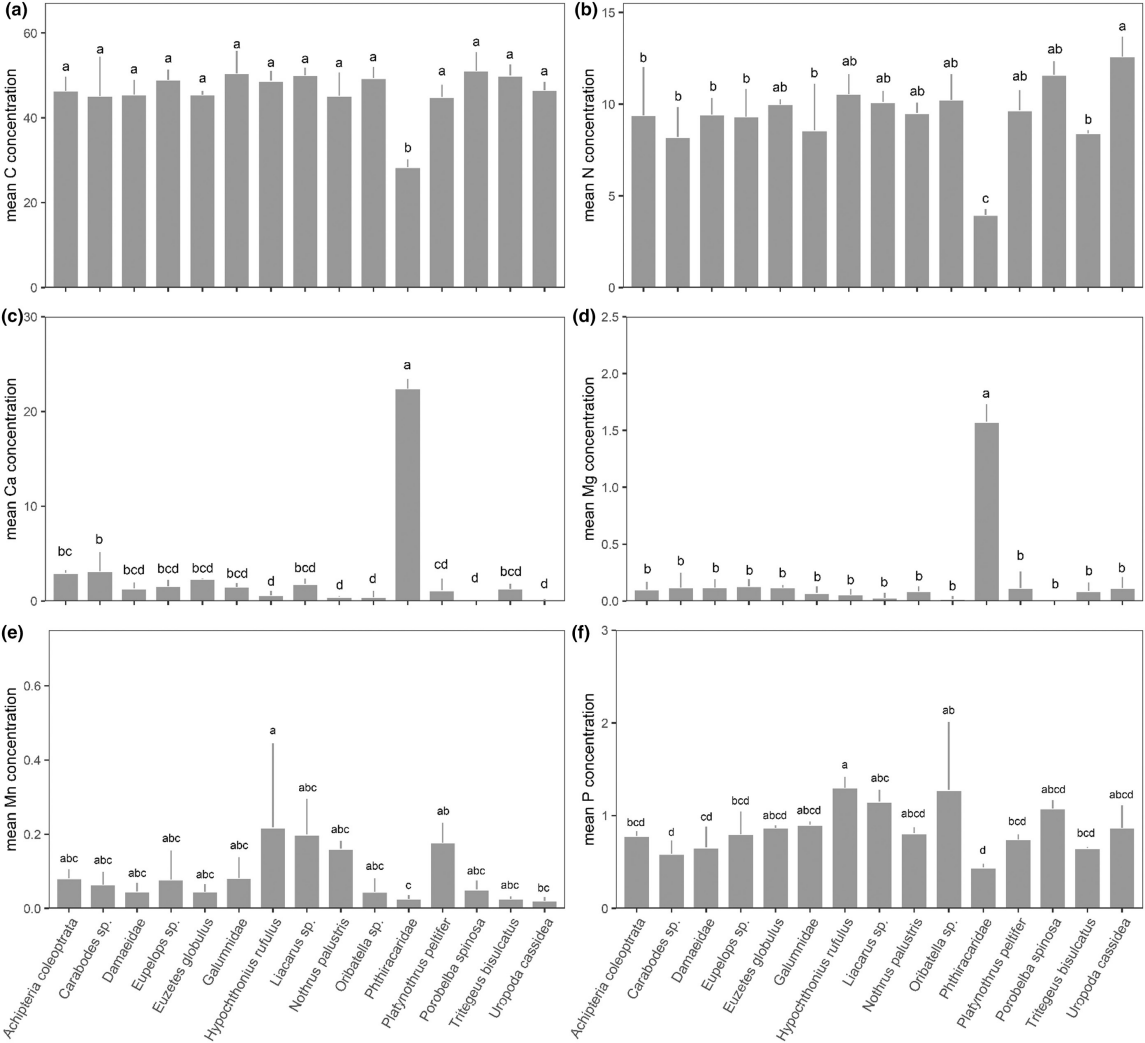
Mean (and standard deviation) concentration of the six elements ((a) C, (b) N, (c) Ca, (d) Mg, (e) Mn, (f) P) in the 15 studied mite taxa which differed significantly among the taxa.

The stoichiometry of the 15 mite taxa differed significantly between species/taxa (MANOVA; Pillai's trace = 1.68, approx. *F*_56,248_ = 3.19, *p* < .001; Figure 1; for significant differences between species see Table 1). The differences were due to significant differences in the concentrations of six (C, Ca, Mg, Mn, N, P) of the 12 elements studied. Carbon concentrations were much lower in Phthiracaridae compared to all other mite taxa (ANOVA, *F*_14,62_ = 9.92, *p* = <.001; Figure 2a). Nitrogen concentrations were also lowest in Phthiracaridae but highest in *U. cassidea* (ANOVA, *F*_14,62_ = 9.36, *p* = <.001; Figure 2b). Calcium concentrations were very high in Phthiracaridae, lower in *Carabodes* sp. and *A. coleoptrata*, and lowest in *H. rufulus*, *N. palustris*, *P. spinosa*, and *U. cassidea* (ANOVA, *F*_14,62_ = 218.9, *p* = <.001; Figure 2c). Similarly, magnesium concentrations were higher in Phthiracaridae compared to all other mite taxa (ANOVA, *F*_14,62_ = 102.1, *p* = <.001; Figure 2d). Manganese concentrations were highest in *H. rufulus*, *Liacarus* sp., *N. palustris* and *P. peltifer*, and lowest in *U. cassidea* and Phthiracaridae (ANOVA, *F*_14,62_ = 3.21, *p* = <.001; Figure 2e). Phosphorus concentrations were highest in *H. rufulus* and *Oribatella* sp., and lowest in Phthiracaridae and *Carabodes* sp. (ANOVA, *F*_14,62_ = 5.41, *p* = <.001; Figure 2f).

**TABLE 1:** *p*‐Values of Hotellings T^2^ pairwise comparisons of elemental concentration of soil mite taxa.

	*Achipteria coleoptrata*	*Carabodes* sp.	Damaeidae	*Eupelops* sp.	Galumnidae	*Hypochthonius rufulus*	*Liacarus* sp.	*Nothrus palustris*	*Oribatella* sp.	Phthiracaridae	*Platynothrus peltifer*	*Tritegeus bisulcatus*
*Carabodes* sp.	0.981											
Damaeidae	**0.012**	0.450										
*Eupelops* sp.	**0.046**	0.625	0.781									
Galumnidae	0.375	0.967	0.590	0.088								
*Hypochthonius rufulus*	0.138	0.871	0.418	**0.005**	0.483							
*Liacarus* sp.	0.055	0.543	**0.026**	**0.004**	0.177	0.194						
*Nothrus palustris*	**0.004**	0.566	**0.010**	0.119	**0.047**	0.071	0.585					
*Oribatella* sp.	**0.029**	0.589	0.428	0.060	0.468	0.685	0.192	**0.049**				
Phthiracaridae	**<0.001**	**<0.001**	**<0.001**	**<0.001**	**<0.001**	**<0.001**	**<0.001**	**<0.001**	**<0.001**			
*Platynothrus peltifer*	0.057	0.373	0.029	**0.013**	0.139	0.077	0.232	0.300	0.073	**<0.001**		
*Tritegeus bisulcatus*	**0.048**	0.909	0.976	0.753	0.857	0.450	0.402	0.129	0.464	**<0.001**	0.157	
*Uropoda cassidea*	**<0.001**	**0.047**	**0.032**	**0.005**	0.190	0.088	**0.013**	**0.010**	0.368	**<0.001**	**0.005**	**0.053**

*Note*: Significant values are marked in bold.

**FIGURE 3:**
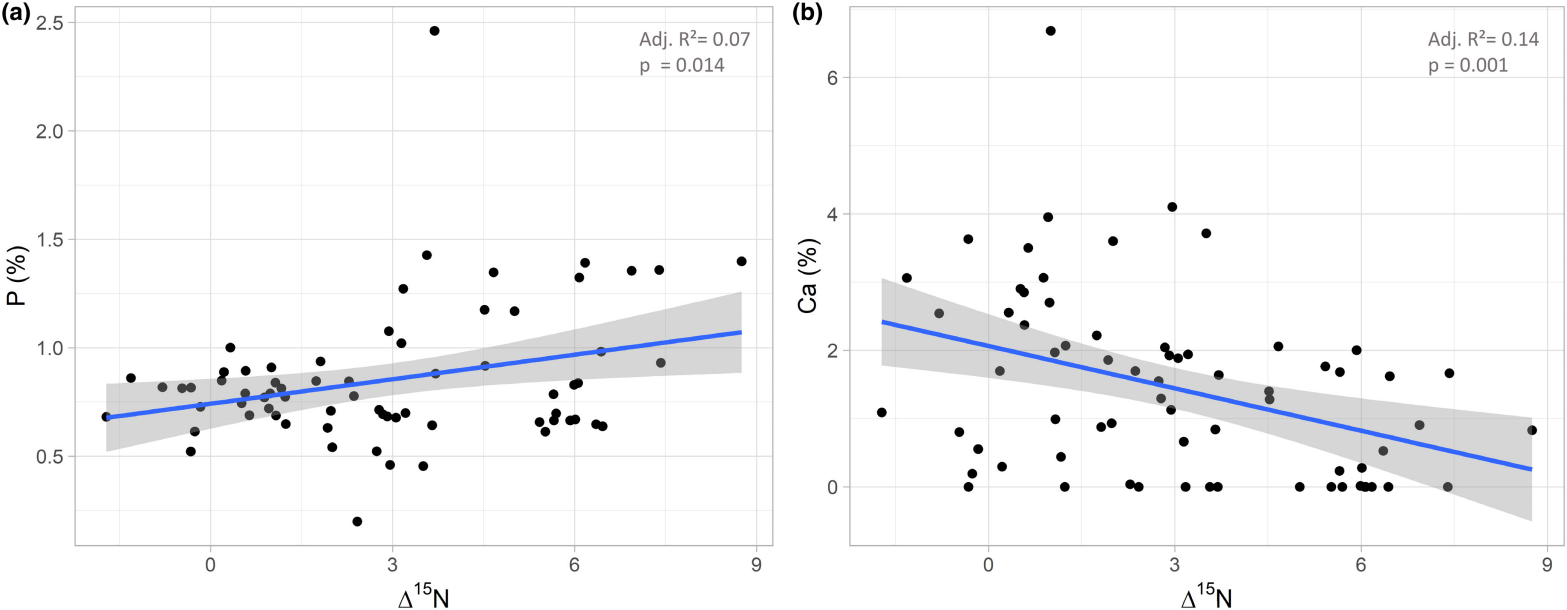
Linear regressions between (a) phosphorus (*y* = 2.32*x* + 0.98) and (b) calcium (*y* = −0.72*x* + 3.99) and Δ^15^N values of the studied mite taxa; Δ^15^N values were calibrated to the litter of the forests they were sampled from, that is, spruce or beech.

**FIGURE 4:**
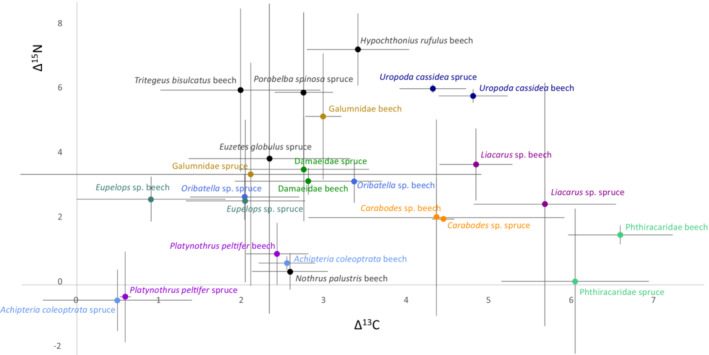
Mean (and standard deviation) Δ^15^N and Δ^13^C values of the investigated mite taxa; Δ^15^N and Δ^13^C values were calibrated to the litter of the forests they were sampled from, that is, spruce or beech.

Δ^15^N values were positively correlated with phosphorus (*t* = 2.53, *p* = .013, adjusted *r* ^2^ = .073; Figure 3a), but negatively with calcium concentrations (*t* = −3.41, *p* = .0011, adjusted *r* ^2^ = .13; Figure 3b; for Δ^15^N (as well as Δ^13^C) values of all studied animal taxa see Figure 4).

## 4 DISCUSSION

We explored the power of stoichiometry for explaining niche differentiation and therefore animal diversity in forest soils using oribatid mites as model organisms. The framework of ecological stoichiometry assumes that organisms face stoichiometric constraints shaping their ecology and distribution (Sterner & Elser, 2002). In the context of this framework, we investigated correlations of the stoichiometry including a wide range of elements and the trophic position (measured by δ^15^N values) of soil mite species of different forest types, i.e. beech and spruce. Although being physically and taxonomically very different, element concentrations of beech and spruce litter did not differ significantly. This may be explained by the fact that both of the investigated forest types stocked on the same parent rock (limestone covered by loess), and this likely resulted in similar element concentrations for tree uptake and thereby in similar litter stoichiometry (Reich & Oleksyn, 2004).

Similar to beech and spruce litter, the stoichiometry of the mite species present in both forests did not differ significantly. On the one hand, this suggests that food resources in both forests provide adequate amounts of nutritional elements for these taxa. On the other hand, it may also indicate that these taxa only allow very limited variations in element ratios supporting the idea of stoichiometric homeostasis (Andrieux et al., 2020; Persson et al., 2010; Sterner & Elser, 2002) and the view that, concerning trophic niches, soil animals have limited ability to respond to changing environmental conditions (Gan et al., 2014; Jochum et al., 2017). The latter is consistent with the rather constant trophic and stoichiometric niche of soil animals across forest ecosystems and soil depths (Lu et al., 2022; Scheu & Falca, 2000; Schneider et al., 2004; Zhang, Chen, Deng, Li, Chen, et al., 2022; Zhang, Chen, Deng, Li, González, & Wang, 2022).

Contrasting the limited variation in element concentrations between forests, element concentrations varied significantly among mite taxa, supporting the existence of multidimensional stoichiometric niches in mite species (González et al., 2017). Overall, the studied mite species differed in six of the studied 12 elements indicating that the stoichiometric niche of soil microarthropods is limited to a narrow spectrum of elements. Phthiracaridae species differed from all other species in high concentrations of Ca and Mg. As shown previously, the high Ca and Mg concentrations result from incorporating calcium carbonate (CaCO_3_) and magnesium carbonate (MgCO_3_) into their cuticle for predator defense (Norton & Behan‐Pelletier, 1991). Calcium concentration was also high in *A. coleoptrata* and *Carabodes* sp., suggesting that these taxa also harden their exoskeleton with CaCO_3_ but to a lesser extent. These taxa predominantly live as primary and secondary decomposers feeding on litter and fungi (Maraun et al., 2011), and this may facilitate the acquisition of Ca from their diet (Cromack et al., 1977), even if Ca is in limited supply (Ott et al., 2014). As a consequence of the high Ca and Mg concentrations, C and N concentrations in Phthiracaridae were low. This resembles diplopods, which also harden their exoskeleton using CaCO_3_ and MgCO_3_ (Thorez et al., 1990; Zhang, Chen, Deng, Li, Chen, et al., 2022; Zhang, Chen, Deng, Li, González, & Wang, 2022). Moreover, Ca concentrations negatively correlated with the trophic level of the studied mite taxa, presumably reflecting more intensive protection against predators of lower trophic level taxa via cuticular hardening.

Nitrogen concentrations were high in *U. cassidea* and intermediate in *A. coleoptrata*, *Carabodes* sp., Damaeidae, *Eupelops* sp., Galumnidae, and *T. bisulcatus*. Nitrogen concentrations typically are high in predatory animals and low in herbivores and decomposers (González et al., 2011), which is related to N concentrations in their diet, i.e. higher N concentrations in animal prey than in plants and litter (González et al., 2011; Sterner & Elser, 2002). This conforms to our data since, as indicated by δ^15^N values, *U. cassidea* (Uropodina; Mesostigmata) lives as predator and the taxa with intermediate N concentrations, except *T. bisulcatus*, live as detritivores or fungivores.

Phosphorus concentrations were high in *H. rufulus* and *Oribatella* sp. High P concentrations typically are related to high energetic demand and turnover (Sterner & Elser, 2002), and this may also apply to *H. rufulus* which, as indicated by δ^15^N values, lives as a predator/scavenger (Maraun et al., 2011). The high P content in *Oribatella* sp., which, according to δ^15^N signatures, lives as secondary decomposer may point to bacterial feeding. Bacteria have high amounts of P in their membrane (Sterner & Elser, 2002), which may be incorporated into the body tissue of bacterivorous mites. Furthermore, high P concentrations in species higher in the food web indicate the accumulation of P by feeding on microbes or other animals (Kaspari, 2021). Generally, the positive correlation between P concentrations and δ^15^N signatures of the mites studied supports our assumption and previous findings (González et al., 2011, 2018) that predators/scavengers are characterized by high P concentration.

High Mn concentrations in *H. rufulus*, *Liacarus* sp., *N. palustris*, and *P. peltifer* also may be related to their diet. As indicated by δ^15^N signatures, *N. palustris*, and *P. peltifer* live as primary decomposers feeding on plant litter, and *Liacarus* sp. lives as a secondary decomposer/fungal feeder (Maraun et al., 2011). Feeding on fungi or litter colonized by fungi may result in high Mn uptake as wood‐colonizing fungi contain high amounts of Mn originating from manganese peroxidase used to decompose lignocellulose (Hofrichter, 2002). In addition, Mn accumulates in wood in the vicinity of fungal hyphae due to the activity of manganese peroxidase (Barnett & Lilly, 1966; Blanchette, 1984). However, as indicated by high δ^15^N signatures (Maraun et al., 2011), *H. rufulus* lives as predator/scavenger suggesting that its high Mn concentration results from high Mn concentrations in its prey/diet which includes dead animals (Riha, 1951).

Results of this study, as well as the studies of Zhang, Chen, Deng, Li, Chen, et al. (2022) and Zhang, Chen, Deng, Li, González, & Wang (2022) on soil macrofauna, indicate that using stoichiometry for analyzing niches of soil animal species is an exciting, but largely unexplored method allowing insight into new niche dimensions. This approach surely can be applied to a wider range of systems and animal taxa (see Zhang, Chen, Deng, Li, Chen, et al., 2022; Zhang, Chen, Deng, Li, González, & Wang, 2022). Coupled with methods allowing to elucidate the trophic position of soil animals, such as the analysis of δ^15^N signatures, or by including other traits of species, the method provides detailed insight into niche differentiation of soil animal species. Furthermore, coupling elementome data with functional and life‐history data may help to test the general validity of the biogeochemical niche hypothesis. In sum, investigating animal stoichiometry may help to better understand the coexistence of soil microarthropod species and thus to solve the enigma of soil animal species diversity (Anderson, 1975).

## AUTHOR CONTRIBUTIONS

**Lara Warnke:** Conceptualization (equal); data curation (equal); formal analysis (equal); investigation (equal); software (equal); visualization (equal); writing – original draft (equal); writing – review and editing (equal). **Dietrich Hertel:** Conceptualization (equal); methodology (equal); resources (equal); writing – review and editing (equal). **Stefan Scheu:** Conceptualization (equal); funding acquisition (equal); project administration (equal); supervision (equal); validation (equal); writing – review and editing (equal). **Mark Maraun:** Conceptualization (equal); formal analysis (equal); methodology (equal); project administration (equal); resources (equal); supervision (equal); validation (equal); writing – review and editing (equal).

## FUNDING INFORMATION

The work has been funded by the German Research Foundation (DFG Priority Program 1374 “Infrastructure‐Biodiversity‐Exploratories,” DFG‐SCHE 376/38‐2).

## CONFLICT OF INTEREST STATEMENT

The authors declare no conflict of interest.

## Supporting information


Figure S1

Figure S2

Table S1

Table S2

Data S1

## Data Availability

Data are available from the Dryad Digital Repository (https://doi.org/10.5061/dryad.dbrv15f61). Supporting information is available as part of the online Appendix.
