# Supplementary material for: Opening up new niche dimensions: The stoichiometry of soil microarthropods in European beech and Norway spruce forests
Source: Ecol Evol. 2023 May 22;13(5):e10122. doi: 10.1002/ece3.10122 (PMC10202621; doi:10.1002/ece3.10122)
Supplement: Supplementary file 1 — Figure S1 [file ECE3-13-e10122-s003.pptx]

## Slide 1
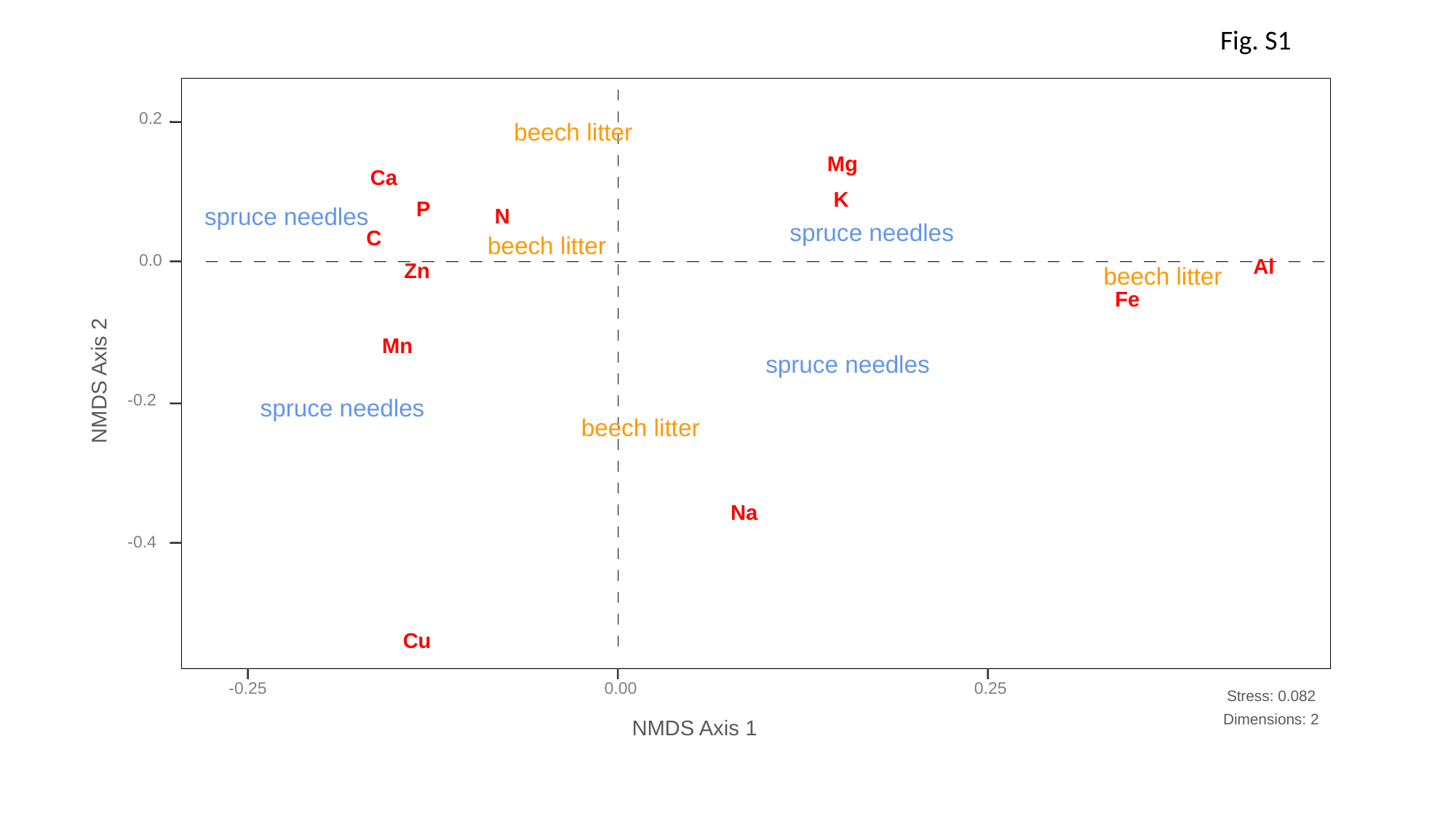

Fig. S1
0.2
beech litter
Mg
Ca
K
P
spruce needles
N
spruce needles
C
beech litter
0.0
Al
Zn
beech litter
Fe
Mn
spruce needles
NMDS Axis 2
-0.2
spruce needles
beech litter
Na
-0.4
Cu
-0.25
0.00
0.25
Stress: 0.082
 Dimensions: 2
NMDS Axis 1
