# Supplementary material for: Opening up new niche dimensions: The stoichiometry of soil microarthropods in European beech and Norway spruce forests
Source: Ecol Evol. 2023 May 22;13(5):e10122. doi: 10.1002/ece3.10122 (PMC10202621; doi:10.1002/ece3.10122)
Supplement: Supplementary file 2 — Figure S2 [file ECE3-13-e10122-s001.pptx]

## Slide 1
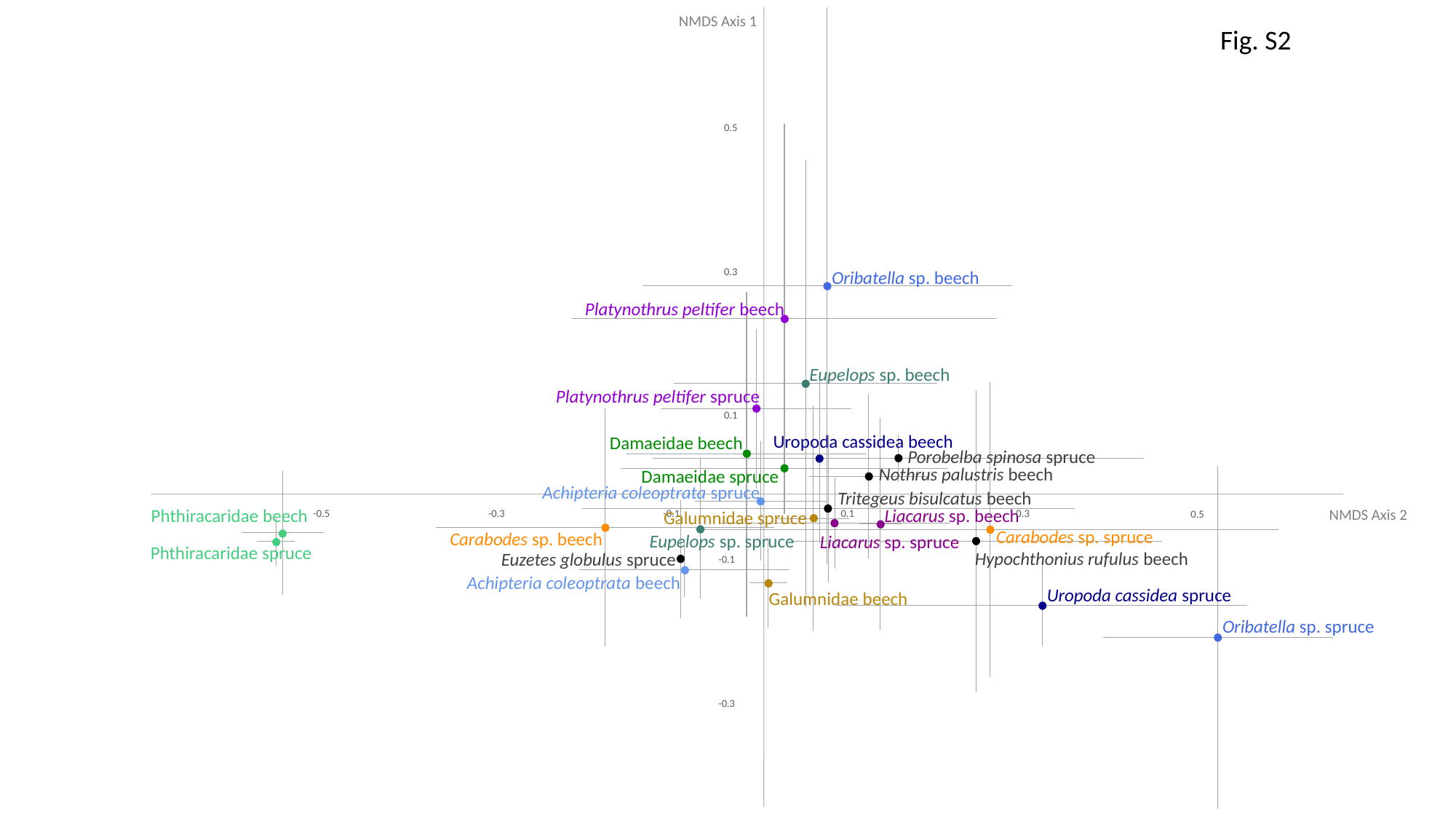

NMDS Axis 1
Fig. S2
0.5
0.3
Oribatella sp. beech
Platynothrus peltifer beech
Eupelops sp. beech
Platynothrus peltifer spruce
0.1
Uropoda cassidea beech
Damaeidae beech
Porobelba spinosa spruce
Nothrus palustris beech
Damaeidae spruce
Achipteria coleoptrata spruce
Tritegeus bisulcatus beech
NMDS Axis 2
Phthiracaridae beech
Liacarus sp. beech
Galumnidae spruce
-0.5
-0.3
-0.1
0.1
0.3
0.5
Carabodes sp. spruce
Carabodes sp. beech
Eupelops sp. spruce
Liacarus sp. spruce
Phthiracaridae spruce
Hypochthonius rufulus beech
Euzetes globulus spruce
-0.1
Achipteria coleoptrata beech
Uropoda cassidea spruce
Galumnidae beech
Oribatella sp. spruce
-0.3
