# Supplementary material for: Opening up new niche dimensions: The stoichiometry of soil microarthropods in European beech and Norway spruce forests
Source: Ecol Evol. 2023 May 22;13(5):e10122. doi: 10.1002/ece3.10122 (PMC10202621; doi:10.1002/ece3.10122)
Supplement: Supplementary file 5 — Data S1 [file ECE3-13-e10122-s002.docx]

**Legends**

Figure S1 Elemental composition of the two forest litter types as reflected by the two axes of non-metric multidimensional scaling (NMDS) of elemental concentrations.

Figure S2 Mean (and standard deviation) stoichiometry of the investigated mite taxa as reflected by the first two axes of non-metric multidimensional scaling (NMDS) of elemental concentrations.

Table S1 Element concentrations of litter and needle samples where animals were taken.

Table S2 Sampling design of the study and distribution of ICP-OES measurements.
